# Cytokine‐ and chemokine‐induced inflammatory colorectal tumor microenvironment: Emerging avenue for targeted therapy

**DOI:** 10.1002/cac2.12295

**Published:** 2022-07-05

**Authors:** Ajaz A. Bhat, Sabah Nisar, Mayank Singh, Bazella Ashraf, Tariq Masoodi, Chandra P. Prasad, Atul Sharma, Selma Maacha, Thasni Karedath, Sheema Hashem, Syed Besina Yasin, Puneet Bagga, Ravinder Reddy, Michael P. Frennaux, Shahab Uddin, Punita Dhawan, Mohammad Haris, Muzafar A. Macha

**Affiliations:** ^1^ Laboratory of Molecular and Metabolic Imaging Cancer Research Department Sidra Medicine Doha 26999 Qatar; ^2^ Department of Medical Oncology Dr. B. R. Ambedkar Institute Rotary Cancer Hospital All India Institute of Medical Sciences (AIIMS) New Delhi 110029 India; ^3^ Department of Biotechnology School of Life Sciences Central University of Kashmir Ganderbal Jammu & Kashmir 191201 India; ^4^ Division of Translational Medicine Research Branch Sidra Medicine Doha 26999 Qatar; ^5^ Genomics Core Facility, QBRI Qatar Foundation Doha 34110 Qatar; ^6^ Department of Pathology Sher‐I‐Kashmir Institute of Medical Sciences Srinagar Jammu & Kashmir 190011 India; ^7^ Department of Diagnostic Imaging St. Jude Children's Research Hospital Memphis TN 38105 USA; ^8^ Center for Advanced Metabolic Imaging in Precision Medicine Department of Radiology Perelman School of Medicine at the University of Pennsylvania Philadelphia PA 19104 USA; ^9^ Academic Health System Hamad Medical Corporation Doha 3050 Qatar; ^10^ Translational Research Institute Hamad Medical Corporation Doha 3050 Qatar; ^11^ Department of Biochemistry and Molecular Biology University of Nebraska Medical Center Omaha NE 68198 USA; ^12^ Laboratory Animal Research Center Qatar University Doha 2713 Qatar; ^13^ Watson‐Crick Centre for Molecular Medicine Islamic University of Science and Technology Awantipora Jammu & Kashmir 192122 India

**Keywords:** chemokine, colorectal cancer, cytokine, drug resistance, epithelial‐mesenchymal transition, immunosuppression, immunotherapy, inflammation, metastasis, tumor microenvironment

## Abstract

Colorectal cancer (CRC) is a predominant life‐threatening cancer, with liver and peritoneal metastases as the primary causes of death. Intestinal inflammation, a known CRC risk factor, nurtures a local inflammatory environment enriched with tumor cells, endothelial cells, immune cells, cancer‐associated fibroblasts, immunosuppressive cells, and secretory growth factors. The complex interactions of aberrantly expressed cytokines, chemokines, growth factors, and matrix‐remodeling enzymes promote CRC pathogenesis and evoke systemic responses that affect disease outcomes. Mounting evidence suggests that these cytokines and chemokines play a role in the progression of CRC through immunosuppression and modulation of the tumor microenvironment, which is partly achieved by the recruitment of immunosuppressive cells. These cells impart features such as cancer stem cell‐like properties, drug resistance, invasion, and formation of the premetastatic niche in distant organs, promoting metastasis and aggressive CRC growth. A deeper understanding of the cytokine‐ and chemokine‐mediated signaling networks that link tumor progression and metastasis will provide insights into the mechanistic details of disease aggressiveness and facilitate the development of novel therapeutics for CRC. Here, we summarized the current knowledge of cytokine‐ and chemokine‐mediated crosstalk in the inflammatory tumor microenvironment, which drives immunosuppression, resistance to therapeutics, and metastasis during CRC progression. We also outlined the potential of this crosstalk as a novel therapeutic target for CRC. The major cytokine/chemokine pathways involved in cancer immunotherapy are also discussed in this review.

**Abbreviations**
APC: adenomatous polyposis coli
CAFs: cancer‐associated fibroblasts
CCL3: C‐C motif chemokine ligand 3
CCR6: chemokine receptor 6
CDH1: cadherin 1
COX‐2: cyclooxygenase‐2
CRC: colorectal cancer
CSF1: colony‐stimulating factor 1
CSF2: colony stimulating factor 2
CTLA‐4: cytotoxic T‐lymphocyte‐associated protein 4
CTLs: cytotoxic T lymphocytes
CXCL: C‐X‐C motif chemokine ligand
CXCR: C‐X‐C chemokine receptor
DCs: dendritic cells
ECM: extracellular matrix
EMT: epithelial‐mesenchymal transition
eNOS: endothelial nitric oxide synthase
FCγR3: immunoglobulin G Fc Receptor 3
FOXP3: forkhead box P3
FRA1: fos‐related antigen 1
GM‐CSF: granulocyte‐macrophage colony‐stimulating factor
GP130: glycoprotein 130
GSK3β: glycogen synthase kinase 3 beta
HMGA1: high mobility group AT‐hook 1
HMGB1: high mobility group box 1
IBDs: inflammatory bowel diseases
ICAM‐1: intercellular adhesion molecule 1
IDO: indoleamine 2,3‐dioxygenase
IL: interleukin
IL‐1β: interleukin‐1 beta
Lin^−^HLA‐DR^−^
: lineage negative‐human leukocyte antigen‐DR isotype
LRG1: leucine rich alpha‐2‐glycoprotein 1
MAPK: mitogen‐activated protein kinase
MCAM: melanoma cell adhesion molecule
M‐CSF: macrophage colony‐stimulating factor
MDSCs: myeloid‐derived suppressor cells
MHC1: major histocompatibility complex class 1
MMP‐9: matrix metalloproteinase‐9
NF‐κB: nuclear factor kappa‐light‐chain‐enhancer of activated B cells
NK: natural killer
OCT4: octamer‐binding transcription factor 4
OS: overall survival
PD‐1: programmed cell death protein 1
PD‐L1: programmed death‐ligand 1
PMN: pre‐metastatic niche
RNS: reactive nitrogen species
RORγt: retinoic‐acid‐receptor‐related orphan nuclear receptor gamma
ROS: reactive oxygen species
RUNX1: runt‐related transcription factor 1
SIPR1: sphingosine‐1‐phosphate receptor 1
SMAD4: SMAD family member 4
SNAI2: snail family transcriptional repressor 2
SNAIL: zinc finger protein SNAI1
SOCS3: suppressor of cytokine signaling 3
SOX2: SRY‐box transcription factor 2
STAT3: signal transducer and activator of transcription 3
TAMs: tumor‐associated macrophages
TCF4: transcription factor 4
TCGA: The Cancer Genome Atlas
TCR: T‐cell receptor
TFAP4: transcription factor activating enhancer‐binding protein 4
Th1: T‐helper 1
TILs: tumor‐infiltrating lymphocytes
TME: tumor microenvironment
TNF‐α: tumor necrosis factor‐α
TRAF6: TNF receptor associated factor 6
Tregs: T‐regulatory cells
TRIM47: tripartite motif containing 47
TSP‐1: thrombospondin‐1
TWIST1: twist‐related protein 1
VEGFA: vascular endothelial growth factor A
ZEB1: zinc finger E‐box‐binding homeobox 1
α‐SMA: alpha‐smooth muscle actin

## 1 BACKGROUND

Colorectal cancer (CRC) is the fourth most common cancer and the third leading cause of cancer‐associated death worldwide [1]. Approximately 25% of patients with CRC have metastases at diagnosis, most commonly liver metastases [2]. Peritoneal cavity metastases develop in approximately 50% of patients treated with chemoradiation therapy [3]. Patients with inflammatory bowel diseases (IBDs), such as ulcerative colitis and Crohn's disease, have an increased risk for developing colitis‐associated CRC [4]. IBDs are characterized by a chronic inflammatory state with an altered intestinal permeability and gut microbiota leading to a dysbiotic intestinal state [5]. These alterations and inflammation alter the normal gut microenvironment and contribute to the pathogenesis of CRC [6, 7]. Unresolved inflammation generates a microenvironment favorable for cellular transformation and the growth of cancer cells. Ulcerative colitis and Crohn's disease account for 10% to 15% of deaths in patients with CRC [8, 9]. In addition, increased consumption of a low‐fiber diet lacking essential nutrients, alcohol/tobacco, red meat, a lack of physical activity [10], and obesity [11] increases the risk of CRC development. Furthermore, family history of CRC, the presence of precancerous CRC polyps, genetic syndromes such as lynch syndrome and familial adenomatous polyposis, and type 2 diabetes are other important risk factors that contribute to the development of CRC [12]. The development of CRC is a multistep process that involves a series of histologic, morphologic, and genetic abnormalities, transforming healthy colorectal epithelial tissues to adenomas that eventually progress into invasive, metastatic, and aggressive tumors. The anomalies driving CRC oncogenesis include somatically altered genetic defects and polymorphisms that affect crucial signaling pathways, such as the Wnt, mitogen‐activated protein kinase (MAPK), Kirsten rat sarcoma viral oncogene homolog (KRAS), transforming growth factor beta (TGF‐β)/bone morphogenetic protein, and DNA damage response pathways [13, 14, 15].

The tumor microenvironment (TME) plays an essential role in cancer initiation, progression, and invasion. The CRC TME, akin to most solid cancers, entails intricate communication between cancer cells and a repertoire of other types of cells, such as cancer‐associated fibroblasts (CAFs), immune cells (e.g., neutrophils and macrophages), and endothelial cells present in the tumor stroma. This cellular communication is mediated by many soluble factors such as cytokines and chemokines, signaling molecules such as growth factors, and extracellular matrix (ECM) components. The balance of pro‐ and anti‐inflammatory cytokines, tumor stage, cytokine receptor expression content, and the stromal cell activation state are determinants of tumor response. Cellular communication among tumor cells, CAFs, and immune cells (i.e., myeloid progenitors/derived suppressor cells) promote an inflammatory TME that fosters CRC tumorigenesis. An elaborate network of deregulated cytokines, chemokines, growth factors, and their receptors underlies this communication.

Persistent exposure of the TME to proinflammatory cytokines supports tumor growth through sustained activation of proinflammatory pathways in CRC. Operating through systemic inflammation, these deregulated pathways strategically reconfigure the local TME, distant vasculature, and pre‐metastatic niche (PMN) to confound antitumor immune responses and induce epithelial‐mesenchymal transition (EMT) in malignant cells, thereby resisting therapeutic responses and facilitating aggressive metastatic CRC development [16, 17]. Notably, specialized cells such as myeloid‐derived suppressor cells (MDSCs), regulatory dendritic cells (DCs), T cells, tumor‐associated macrophages (TAMs), enterocytes, paneth cells, and goblet cells participate in mediating a large proportion of these responses. Studies reporting these responses have also identified various prognostic indicators and therapeutic targets for CRC. This review highlights the collective interplay between different cytokines, chemokines, their cellular effectors, and the TME during CRC oncogenesis and how the inflammatory network (and its cellular effectors) may be targeted to improve therapeutic outcomes.

## 2 CYTOKINES AND CHEMOKINES INDUCE INFLAMMATION IN CRC

Chronic inflammation is a crucial hallmark of many cancers, including CRC [18], and the immunologic TME plays a decisive role in developing CRC [14, 19‐21]. Inflammatory immune cells and associated cytokines in the TME are thought to play a double‐faced role in the initiation and progression of CRC. Even the adaptive immune system is believed to represent a double‐edged sword by being involved in either immune surveillance or tumor‐promoting inflammation, which is crucial in determining the progression of CRC [22]. For example, interleukin‐17 (IL‐17) is a proinflammatory cytokine that can promote tumor‐elicited inflammation and help cancer cells to escape immune surveillance [23]. Also, tumor‐elicited inflammation driven by the activation of IL‐23/IL‐17 promotes colorectal tumorigenesis [24, 25]. Increased IL‐23, IL‐23 receptor, and IL‐17A levels are associated with a poor prognosis and rapid development of metastatic disease in CRC patients [26]. The local host inflammatory response, measured by the density of tumor‐infiltrating lymphocytes (TILs), confers a substantial positive predictive value [27, 28] and is associated with better CRC outcomes [29]. For decades, many investigations have identified and characterized the factors driving intestinal inflammation‐mediated CRC tumorigenesis. Besides the contribution of diet and gut microbiota to CRC oncogenesis, these studies revealed that deregulated cytokines, chemokines, growth factors, and matrix‐remodeling enzymes at the core of intestinal inflammation facilitate CRC development [30].

**FIGURE 1:**
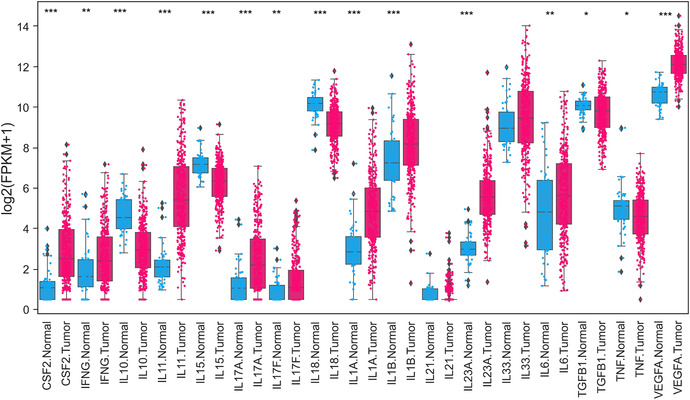
Expression profile of cytokines and chemokines in CRC. Differentially expressed cytokines comparing normal solid tissues (blue color) with primary tumors (red color) of COADREAD. Expression data of normal and cancer tissues for TCGA or GDC was downloaded from University of California, Santa Cruz (UCSC) Xena (https://xenabrowser.net/) genomics explorer. A total of 431 samples data of COADREAD was available for comparison, including 380 tumors and 51 normal solid tissues. Boxplots were created, and expression values were log‐transformed for presentation and comparison. *P* values were assessed using Mann‐Whitney *U* Test. *P < 0.05; **P <0.01; ***P <0.001. Abbreviations: CRC, colorectal cancer; TCGA, The Cancer Genome Atlas; COADREAD, Colorectal adenocarcinoma Rectal adenocarcinoma; GDC, Genomic Data Commons; CSF, colony‐stimulating factor 2; IFG, interferon gamma; IL, interleukin; TGF, tumor growth factor; TNF, tumor necrosis factor; VEGFA, vascular endothelial growth factor A; FPKM, fragments per kilobase of transcript per million mapped reads

In response to localized inflammation, cytokines, such as tumor necrosis factor‐α (TNF‐α), IL‐1β, and IL‐6 produced by gut immune cells [14, 31], stimulate complex crosstalk among the gut microenvironment cells, including enterocytes, paneth cells, goblet cells, and macrophages, that drive chronic inflammation in IBDs such as ulcerative colitis and Crohn's disease [32]. TNF‐α and IL‐6 are central players in developing CRC via activation of the critical oncogenic transcription factors nuclear factor kappa‐B (NF‐κB) and signal transducer and activator of transcription 3 (STAT3), respectively [33, 34]. IL‐6 and TNF‐α synergistically activate STAT3 and NF‐κB to promote CRC cell growth in vivo [35]. TNF‐α signaling has many components, including soluble or membrane‐bound ligands and TNF receptors (TNF‐αR1 and TNF‐αR2). The binding of soluble TNF‐α to TNF‐αR1 activates NF‐κB, a crucial transcription factor controlling many inflammatory pathways [36, 37, 38, 39]. IL‐6 secreted in the CRC stroma by the lamina propria, T cells, macrophages, and CAFs [40] is considered a master pro‐inflammatory cytokine promoting chronic inflammation, the antiapoptotic phenomenon [34, 41, 42], and CRC progression [43, 44, 45]. Increased serum IL‐6 [46, 47, 48, 49] and TNF‐α levels [50, 51, 52] are associated with advanced‐stage liver metastasis and short overall survival (OS) of patients with CRC. Increased *TNF* mRNA expression also occurs in CRC tumor xenografts [53]. On the contrary, few studies have reported the anti‐tumor activity of TNF‐α [54, 55]. A study reported that the secretion of TNF‐α by J2T12 cells (plasmacytoma J558 cell‐derived clone expressing TNF‐α) suppressed tumor growth in vivo when injected into the syngeneic BALB/c mice, whereas the inoculation with parental J558L cells resulted in the development of tumors, suggesting that the anti‐tumor activity of TNF‐α possibly via recruitment of inflammatory cells such as macrophages, neutrophils, and natural killer (NK) cells [54]. Another study reported that the production of lipopolysaccharide‐induced TNF‐α was reduced in CRC patients compared to healthy controls [56]. While another study reported lower TNF‐α levels in the tumors and higher interferon‐gamma (IFN‐γ) levels in the peripheral blood mononuclear cells of CRC patients [57]. Evans *et al*. [55] observed a worse survival for the patients expressing lower levels of TNF‐α and IFN‐γ. Significant lower expression of TNF‐α in CRC tumors compared to normal tissues was also observed in the Cancer Genome Atlas (TCGA) data (**Figure** 1).

**FIGURE 2:**
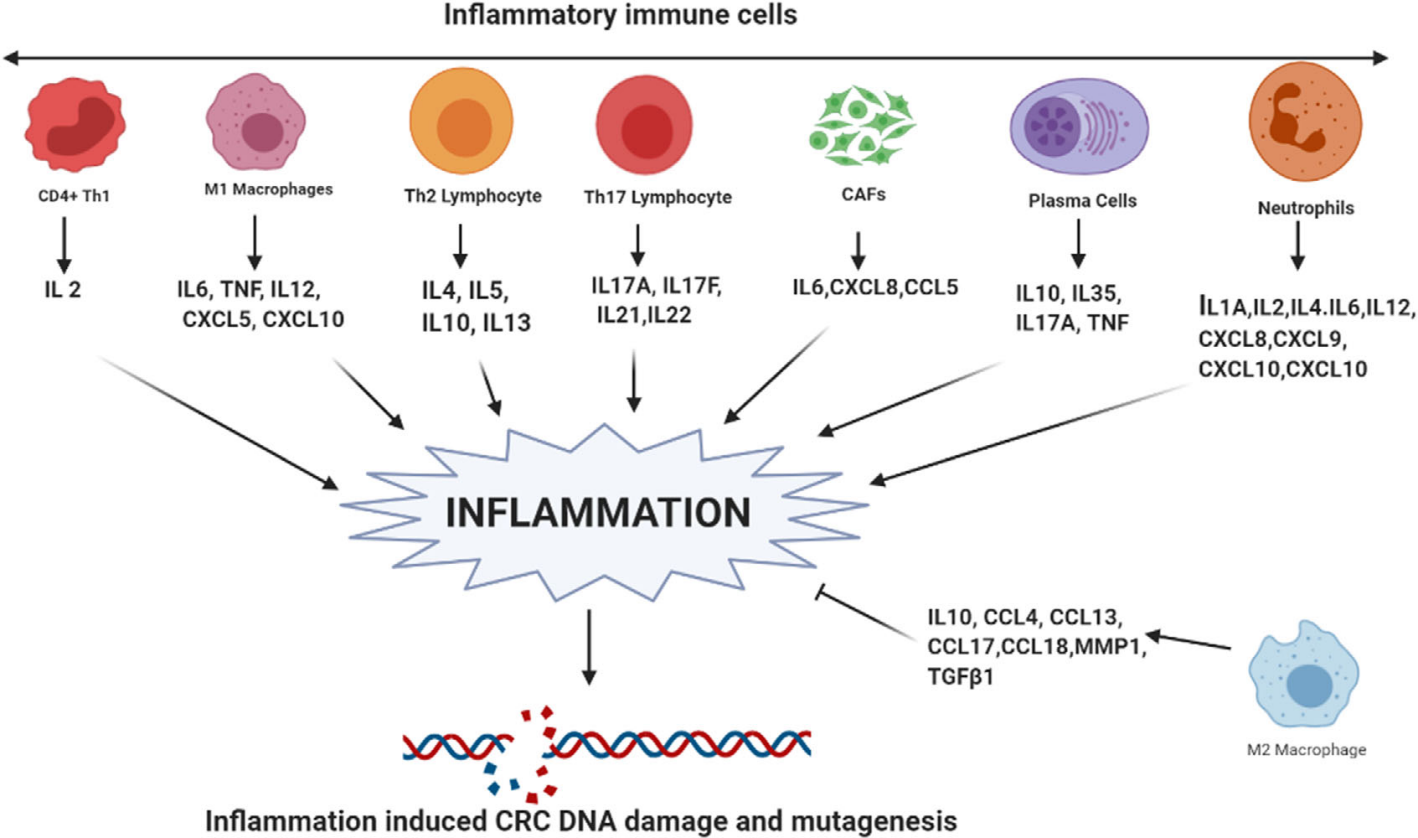
Cytokines and chemokines as inducers of inflammation. Different immune components, including Th1 cells, M1 macrophages, Th2 lymphocytes, Th17 lymphocytes, CAFs, plasma cells, and neutrophils, are involved in inflammation in CRC by secreting different interleukins and chemokines. However, M2 macrophages oppose this inflammation. The collective interplay of these cytokines determines the inflammatory microenvironment in CRC, which leads to increased ROS‐ and other inflammatory radical‐mediated DNA damage and mutagenesis in CRC. **Abbreviations**: CRC, colorectal cancer; CAFs, cancer‐associated fibroblasts; Th, T helper cells; ROS, reactive oxygen species

Expression analysis of different cytokines downloaded from TCGA demonstrated significant differential expression (**Figure** 1), with the majority showing overexpression (colony‐stimulating factor 2 [*CSF2*]*, IFNG, IL‐11, IL‐17A, IL‐17F, IL‐1A, IL‐1B, IL‐21, IL‐23A, IL‐33, IL‐6*, vascular endothelial growth factor A [*VEGFA*]) and a few down‐regulated (e.g., *IL‐10, IL‐15, IL‐18, TGF‐β1, TNF*). Similar to TNF‐α and IL‐6, cytokines such as IL‐11, IL‐17A, and IL‐22 are overexpressed at the mRNA and protein levels in CRC [58, 59, 60, 61] and facilitate human and mouse CRC development [34]. TNF‐α, IL‐4, IL‐10, IL‐17A, and IL‐17F levels are also elevated in the sera of patients with advanced‐stage CRC [62]. IL‐17A‐producing CD4**^+^** T cells and those co‐expressing RAR‐related orphan receptor gamma t (RORγt) and forkhead box P3 (FOXP3) proteins are also abundant in human CRC tumors [63]. In contrast with TNF‐α and IL‐6, IL‐17A, IL‐17F, and IL‐22 exhibit antitumor effects by enhancing immune cell recruitment and tissue repair and by inhibiting inflammation and proangiogenic factors in CRC [64]. In addition, circulating proinflammatory cytokines, including IL‐8, IL‐2, and IL‐6, are elevated in patients with CRC [65]. Moreover, IL‐1β and C‐C motif chemokine ligand 3 (CCL3) levels are increased in the feces and associated with lesion severity in *Helicobacter bilis*‐infected mothers against decapentaplegic homolog 3**^–/–^** (SMAD3**^–/–^**) mice, an inflammation‐associated colon cancer model [66]. Cytokines, such as IFN‐γ, IL‐15, and IL‐18, inhibit CD8**^+^** cytotoxic T lymphocyte (CTL)‐mediated antitumor immune responses [34]. In addition, many pro‐ and anti‐inflammatory cytokines and chemokines, such as CCL2, C‐X‐C motif chemokine ligand 8 (CXCL8), CXCL2, CSF1, and CSF2 (also termed granulocyte‐macrophage [GM]‐CSF), are produced by either neoplastic or stromal cells in the TME (**Figure** 2).

Prostaglandin E2 (PGE2) is produced by cancerous stromal cells during tumor progression that acts via both autocrine and paracrine mechanisms and exerts its function through ligation with E‐type prostanoid (EP) receptors 1–4 (EP 1–4) [67]. PGE2 and the corresponding enzyme involved in its synthesis, cyclooxygenase 2 (COX2), are overexpressed in CRC [68, 69] and are found to promote tumor cell growth through the growth factor signaling activation, resistance to apoptosis, and induction of angiogenesis [70]. PGE2 further modulates TME by suppressing antitumor immune responses and enhancing tumor immune evasion, thereby giving rise to more aggressive tumors [71]. Non‐steroidal anti‐inflammatory drugs and unselective COX inhibitors such as aspirin are found to inhibit PGE2 production and have been associated with a reduced risk and recurrence of CRC [72, 73, 74], which further questions that how modification of CRC TME can give rise to a favorable outcome in patients with CRC. The different TME components and their involvement in inflammatory cytokine and chemokine production, facilitating CRC oncogenesis, are shown in **Table** 1.

**TABLE 1:** Inflammatory cytokines/chemokines and their functions in the TME

Cell type in TME	Cell subtype	Inflammatory cytokines/chemokines	Functions	Reference
**Inflammatory cells**	M1 macrophage	IL‐6, TNF‐α, IL‐12A, IL‐12B, IL‐23A, CXCL5, CXCL9, CXCL10, CXCL11	Activation of inflammation	[281]
	M2 macrophage	IL‐10, CCL4, CCL13, CCL17, CCL18, MMP1, TGF‐β1	Resolution of inflammation	[282]
	Th1 lymphocyte	IFN‐γ, IL‐2	Activation of cytotoxic immune response	[283]
	Th2 lymphocyte	IL‐4, IL‐5, IL‐10, IL‐13	Activation of humoral immune response	[283]
	Th17 lymphocyte	IL‐17A, IL‐17F, IL‐21, IL‐22	Activation of neutrophils	[283]
	Treg lymphocyte	TGF‐β	Immunosuppression	[283]
	Plasma cell	IL‐10, IL‐35, TNF‐α, IL‐17A, CSF2	Both pro‐ and anti‐inflammatory mediators	[284]
	Neutrophil	IL‐1A, IL‐1B, IL‐1RA, IL‐6, IL‐12 CXCL8, CXCL9, CXCL10, CXCL11, CCL2, CCL3, CCL4, TGF‐β1, VEGF‐A	Activation of inflammation; depending on the type of polarization, also anti‐inflammatory mediators are secreted	[285]
	Eosinophil	IL‐1A, IL‐2, IL‐4, IL‐6, IL‐12, CXCL1, CXCL8, CXCL10, CCL3, CCL5, CCL11	Th2 type immune responses	[286]
	Myeloid derived suppressor cell	IL‐10, TGF‐β	Immunosuppression	[287]
	Mast cell	IL‐4, IL‐5, IL‐6, TNF‐α, CSF2	Th2 type immune responses	[288]
**Stromal cells**	Cancer associated fibroblast (CAF)	HGF, IL‐6, SDF‐1, TGF‐β, IL‐8, IL‐13, CXCL12, CXCL14, VEGF	Inflammation, immune suppression	[289]
	Tumor associated endothelial cells (TEC)	VEGF superfamily, TNF‐α, INF‐γ, CXCL12, CXCL13, CXCL10/11, IL‐6, IL‐7, IL‐8, CCL19/21	Angiogenesis, inflammation, immune responses	[290]
**Cancer cells**	Cancer cells, neoplastic cells	IL‐6, CSF1, CSF2, CCL2, CXCL1, CXCL8, CXCL10, CXCL12, VEGF‐A	Inflammation, immune cells recruitment, angiogenesis	[16]

Abbreviations: IL, interleukin; CXCL, C‐X‐C motif ligand; TNF‐α, tumor necrosis factor‐alpha; TGF‐β, transforming growth factor‐beta; TGF‐β1, transforming growth factor‐beta 1; IFN‐γ, interferon‐gamma; VEGF, vascular endothelial growth factor; VEGF‐A, vascular endothelial growth factor A; CSF1, colony stimulating factor 1; CSF2, colony stimulating factor 2; MMP, matrix metalloproteinase; CCL, chemokine ligand; IL‐1R, interleukin‐1 receptor; HGF, hepatocyte growth factor.

Although intense local inflammation is associated with antitumor immune responses, systemic inflammation is associated with protumor immune response, poor prognosis [75], and reduced OS in advanced CRC [76, 77, 78]. Systemic inflammation elicits a favorable TME for tumor initiation and progression, angiogenesis, increased vascular permeability, cancer cell infiltration [75], and PMN development, thereby promoting distant metastases [79]. Although the major driving factors of systemic inflammation responses are complicated and elusive [80], its hallmarks in CRC include elevated proinflammatory cytokines and acute‐phase proteins that enter the blood circulation [16]. These factors alter the interactions between neoplastic and non‐neoplastic cells in the TME and promote CRC progression [16]. Furthermore, CRC systemic inflammation is associated with increased serum IL‐6 and CXCL8 levels [81, 82] and has emerged as an important marker of patient prognosis [83, 84]. The Glasgow prognostic score, modified Glasgow prognostic score, and platelet lymphocyte ratio are routinely used to monitor CRC progression; however, inflammatory cytokine levels are now being investigated as predictors of response to targeted therapies. Guinney *et al*. [85] proposed a classification system that includes four consensus molecular subtypes (CMSs), each possessing a distinct feature: CMS1 (microsatellite instability immune, 14%) is characterized by a hypermutated, microsatellite unstable and strong immune activation phenotype; CMS2 (canonical, 37%), an epithelial subtype marked with activated WNT and MYC signaling; CMS3 (metabolic, 13%), an epithelial subtype with prominent metabolic dysregulation; and CMS4 (mesenchymal, 23%) is characterized by prominent activation of TGF‐β, stromal invasion, and angiogenesis.

## 3 CYTOKINES AND CHEMOKINES MODULATE CRC MICROENVIRONMENT

**FIGURE 3:**
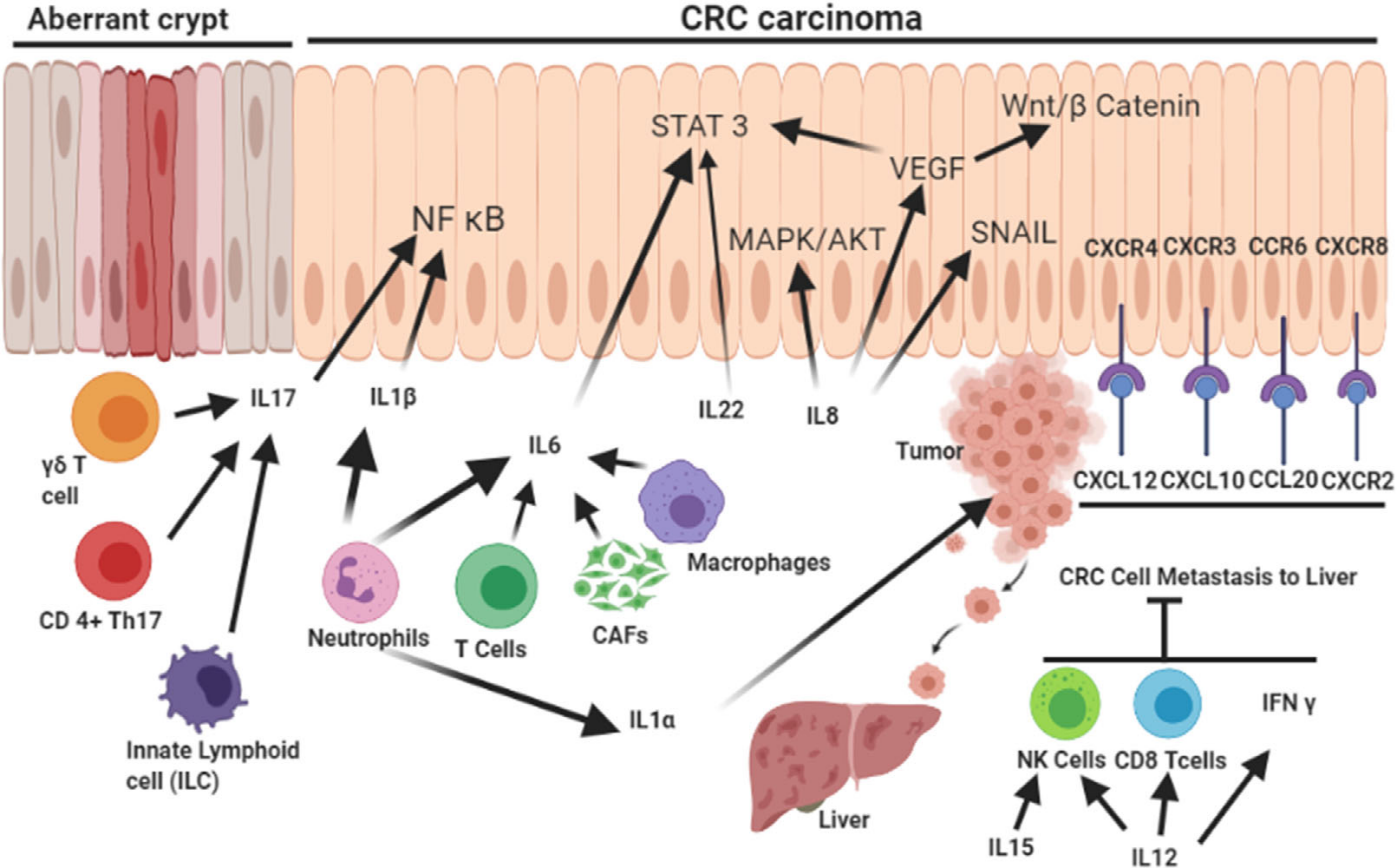
Cytokines and chemokines as modulators of the TME. Different immune cell types, such as neutrophils, T‐cell subtypes, macrophages, CAFs, and innate lymphoid cells, play distinct roles in the secretion of different interleukins, contributing to the activation of inflammatory NF‐κB, proliferative STAT3/MAPK/AKT, and proangiogenic VEGF and SNAI1, which induces EMT. The different chemokine receptor‐ligand pairs support CRC cell metastasis in the liver, whereas some interleukins (IL‐15 and IL‐12) inhibit metastasis and act as antitumor cytokines. **Abbreviations**: CAFs, cancer‐associated fibroblasts; NF‐κB, nuclear factor kappa B; VEGF, vascular endothelial growth factor; EMT, epithelial to mesenchymal transition; CRC, colorectal cancer; SNAIl, snail family transcriptional repressor 1; IL, interleukin; STAT3/MAPK/AKT, signal transducer and activator of transcription 3/ mitogen‐activated protein kinase/protein kinase B

The CRC TME is subjected to the mutual interplay between pro‐ and anti‐inflammatory cytokine networks (**Figure** 3). Several cytokines and chemokines and their cognate receptors are upregulated in CRC and are often associated with poor patient prognosis. For instance, the CXCL12 chemokine and its receptor C‐X‐C chemokine receptor type 4 (CXCR4) are associated with CRC progression [86, 87]. Indeed, activation of the CXCL12/CXCR4 axis is related to infiltration of pro‐tumorigenic CD206^+^ TAMs at the invasive front and liver metastasis in CRC specimens. Mechanistically, CXCL12/CXCR4 activation upregulates several microRNAs that are transferred to TAMs through CRC‐derived extracellular vesicles [88]. TAMs stimulate immunosuppression, angiogenesis, and priming of the PMN, thereby promoting tumor growth and metastasis. However, TAMs exhibit invasive behaviours and EMT in CRC cells [89, 90], and high TAM density is generally associated with a worse prognosis except for CRC, where it is associated with improved survival [91, 92, 93], possibly due to increased chemotherapeutic response. Studies on CRC have shown that the association between high TAM densities and better survival in patients with stage III CRC is the outcome of their interaction with 5‐fluorouracil [94]. Synergism with 5‐fluorouracil likely accounts for the entire prognostic value of TAMs in CRC [94]. Preclinical studies in different cancers have also demonstrated a complex dual role for TAMs in modulating the response to anticancer therapies [95]. Similar to TAMs, CAFs have also been shown to stimulate cancer cell growth and tumor angiogenesis by enhancing stromal cell derived factor‐1 (SDF‐1)/CXCL12 signaling in CRC [96].

CXCR3 and its corresponding ligands CXCL9 and CXCL10 are elevated in CRC and associated with tumor metastasis [97, 98]. Toiyama *et al*. [99] used cytokine arrays to investigate cytokine profiles in the sera of patients with stage IV CRC and compared them with those of patients with stage I CRC. Serum CXCL10 levels are highly associated with pathologic tumor stage, vascular invasion, and distant metastases. Moreover, elevated serum CXCL10 levels were an independent marker of liver metastasis [99]. Another chemokine/receptor axis, CCL20, and its cognate receptor chemokine receptor 6 (CCR6) are upregulated in several cancers. These cognate receptors are elevated in both CRC tumors and liver metastases compared with non‐transformed tissues [100]. CCR6 stimulation by CCL20 promotes the proliferation and migration of CRC in vitro and in vivo [101, 102]. Lee *et al*. [103] evaluated the effects of tumor‐secreted IL‐8 and its receptor CXCR2 on CRC progression and metastasis. Increased levels of IL‐8 in the TME promoted the growth and metastasis of human and mouse colon cancer cells.

More recently, CAF by secreting Chitinase 3‐like 1 (CHI3L1) has been shown to induce IL‐8 secretion, thereby promoting angiogenesis in CRC [104]. In contrast, *CXCR2* deletion reduced tumor angiogenesis and enhanced tumor necrosis [103]. Interestingly, the orally active antagonists SCH‐527123 and SCH‐479833, which target CXCR2 and CXCR1, prevent neovascularization and increase apoptosis, thereby inhibiting CRC liver metastasis [105].

The IL‐1 cytokine family comprises 11 members, which play an essential role in T‐cell activation [106]. IL‐1β, which is upregulated in CRC, is a potent activator of NF‐κB and is involved in CRC progression [60]. Tu *et al*. [107] demonstrated that stomach‐specific expression of human IL‐1β protein in a transgenic mouse model led to spontaneous gastric inflammation and recruitment of MDSCs. IL‐1β activates MDSCs in vitro and in vivo through an IL‐1RI/NF‐κB pathway, leading to cancer development [107]. Similarly, neutrophil infiltration is associated with intestinal inflammation and CRC development. IL‐1β is secreted by infiltrating neutrophils and induces IL‐6 production by intestinal mononuclear phagocytes, promoting tumorigenesis in colitis‐associated cancer mouse models [108]. CRC cell‐derived IL‐1α augments angiogenesis by modulating stromal cells within the TME and facilitates metastases to distant organs, including peritoneal and liver metastases [109]. While the role of CAFs in CRC has been extensively reviewed [110, 111], Heichler *et al*. [112] showed that IL‐6 and IL‐11 are frequently upregulated in CRC, activate STAT3 in CAFs, and are associated with poor survival. Indeed, they reported that constitutive activation of STAT3 in colon fibroblasts promotes tumorigenesis in an experimental model of CRC. In addition, CAFs was shown to induce metastasis by secreting leucine rich alpha‐2‐glycoprotein 1 (LRG1) via the IL‐6/STAT3 signaling pathway [113]. Furthermore, by upregulating inflammatory mediators such as TNF‐α, TGF‐β, chemokines, CXCL2 and SDF‐1, IL‐1β, CAFs induce CRC invasion and metastasis in vitro [114].

Besides regulating cytokines and chemokines, CAFs also promotes invasion, metastasis and CRC progression by regulating circEIF3K‐miR‐214 in vitro [115]. Similarly, CAF‐secreted exosome‐mediated miR‐21 was shown to induce invasion in CRC cells [116]. CAFs also secrete WNT2 and promote migration and invasion of CRC cells [117, 118]. In collaboration with this study, earlier studies also reports that CAF‐released WNT2 promote CRC metastasis by muffing CD differentiation by stimulating the production of suppressor of cytokine signaling 3 (SOCS3) in DC precursors [119].

In contrast, the IL‐12 family suppresses tumor growth by inducing the expansion of T‐helper 1 (Th1) responses and activation of cytotoxic immune effectors, such as NK cells and CTLs [120]. IL‐12 also induces IFN‐γ production in these cells, inhibiting metastasis and tumor growth [120, 121]. Similarly, IL‐15 exhibits antitumor properties in CRC. Although produced by many cells within the TME, IL‐15 promotes Th1 cell‐mediated immunity, provides costimulatory signals to effector CTLs, and activates NK cells [122, 123, 124]. Moreover, patients with CRC with genomic deletion of *IL‐15* have reduced lymphocyte proliferation and a higher risk of tumor relapse than patients without *IL‐15* deletion [125].

Unlike a healthy microenvironment, the TME is predominated by multiple immunosuppressive cytokines. In CRC, the TME‐mediated immunosuppressive cytokines, such as TGF‐β, VEGF, IL‐6, CXCL3, CXCL4, and high mobility group box 1 (HMGB1), play a key role in immune evasion mechanisms [126]. TGF‐β is a well‐known immunomodulator that regulates TME in favor of cancer cells [127]. Additionally, another multifunctional cytokine VEGF also acts as a modulator of immune suppression in the TME of CRC. VEGF also mediates immunosuppression in CRC by inhibiting T‐cell function, which facilitates recruitment of T‐regulatory cells (Tregs) and MDSCs, inhibits the activation and differentiation of DCs from a CD34**^+^** precursor in the TME, and suppresses NF‐κB activation in the hematopoietic progenitor cells [128]. Together, these findings suggest that TGF‐β and VEGF are the dominant immunosuppressive factors involved in CRC progression. Moreover, IL‐6, a multipotent immunosuppressor that is expressed in DCs, facilitates immune evasion of tumor cells in the CRC TME via complex signaling pathways [129]. Chemokines CXCL3 and CXCL4 act as immunosuppressive modulators in the CRC TME via regulating Tregs and CTLs [130]. HMGB1 has an important role in cancer progression as it acts as a potential immunosuppressive mediator in the TME by inducing apoptosis of macrophage‐derived DCs and promotes metastasis in CRC [131, 132].

The IL‐17 family comprises six subtypes of cytokines, in which IL‐17A plays a protumorigenic role, whereas IL‐17F exhibits an antitumorigenic function [61, 133]. CD4**^+^** Th17 immune cells are the primary source of IL‐17A in the CRC TME [134], but other mediators include CTL subsets, γδ T cells, and innate lymphoid cells [135, 136]. IL‐17 serum levels are found to be elevated in patients with CRC, and the expression of IL‐17 has been found to be associated with tumor size and tumor stage [137]. Similarly, patients with enhanced expression of the genes associated with a Th17 signature have a poor CRC prognosis [138]. In contrast with IL‐17A, IL‐17F exerts an inhibitory function in colonic tumorigenesis. *IL‐17F‐*deficient mice develop larger intestinal tumors than do wild‐type control mice in a colitis‐associated cancer murine model [61]. Overexpression of *IL‐17F* in HCT116 CRC cells also inhibits angiogenesis and reduces tumor growth [61].

IL‐33, a member of the IL‐1 superfamily, is a proinflammatory cytokine that is expressed by the epithelial and endothelial cells (ECs) [139]. While ECs are present in the blood and lymphatic vessels, and lining their innermost layer promotes selective permeable exchange between the blood and tissue, tumor‐associated ECs (TECs) have been shown to secrete many soluble factors, also called “angiocrine” [VEGFA, basic fibroblast growth factor (FGF), platelet‐derived growth factor (PDGF), Jagged ½, and nitric oxide (NO), E‐selectins, intercellular adhesion molecule 1 [ICAM‐1], and vascular cell adhesion molecule 1 (VCAM‐1)], to promote tumorigenicity and therapeutic resistance in many cancers including CRC [140]. Through VEGF‐C/VEGFR3 signalling pathway activation, TECs reduced lymphatic endothelial barrier integrity and promoted lymphangiogenesis and metastasis in CRC in vivo [141]. Furthermore, by secreting CCL5, TECs are known to promote metastasis [142]. By secreting IL‐33, TECs promote neovascularization and CRC metastasis to liver [143]. In addition to angiogenesis, TECs are known to modulate immune systems and help CRC cells to escape immunity. IL‐33 plays an important role in the regulation of immune responses to pathogens and allergens and has been associated with IBDs [144]. The mRNA and protein expression of IL‐33 has been found to be significantly elevated in the inflamed mucosa of ulcerative colitis and contributes to the development of chronic inflammation [145]. Increased expression of IL‐33 and suppression of tumorigenicity 2 (ST2) has been reported in colorectal adenomas and CRC tissues compared to healthy tissues [146]. Despite significant evidence of pro‐tumorigenic role of TECs secreted IL‐33 in CRC by inhibiting anti‐tumor immunity, remodelling tumor stroma, and enhancing angiogenesis [147], few studies have reported the anti‐tumorigenic effect of IL‐33 in CRC as well [148, 149, 150].

The findings mentioned in this section and at other places in the review indicate that inflammatory TME is driven by proinflammatory cytokines and chemokines either secreted by tumor cells themselves or by additional cells recruited to the TME. These inflammatory cytokines and chemokines support the growth and differentiation of tumor cells, impart inflammation to the TME, and fuel drug resistance, thus representing a viable therapeutic strategy [151, 152]. Moreover, CRC is characterized by different molecular subtypes, thus resulting in more tumor heterogeneity. Various treatments have been described for different types of CRC tumors based on ptheir molecular subtype, and many therapeutic agents designed to target tumor stroma are currently in clinical trials [153]. Thus, targeting the inflammatory TME in CRC clinically can be effective as integration of data on TMEs with genome and transcriptome profiles might identify the best therapeutic combinations for each patient's tumor type and also open a way for personalized medicine. In this direction, several proinflammatory cytokines such as IL‐6, IL‐1β, and TNFα or their downstream effector molecules are evolving as prospective drug targets for anticancer therapy.

## 4 CYTOKINES AND CHEMOKINES INDUCE EMT IN CRC

**FIGURE 4:**
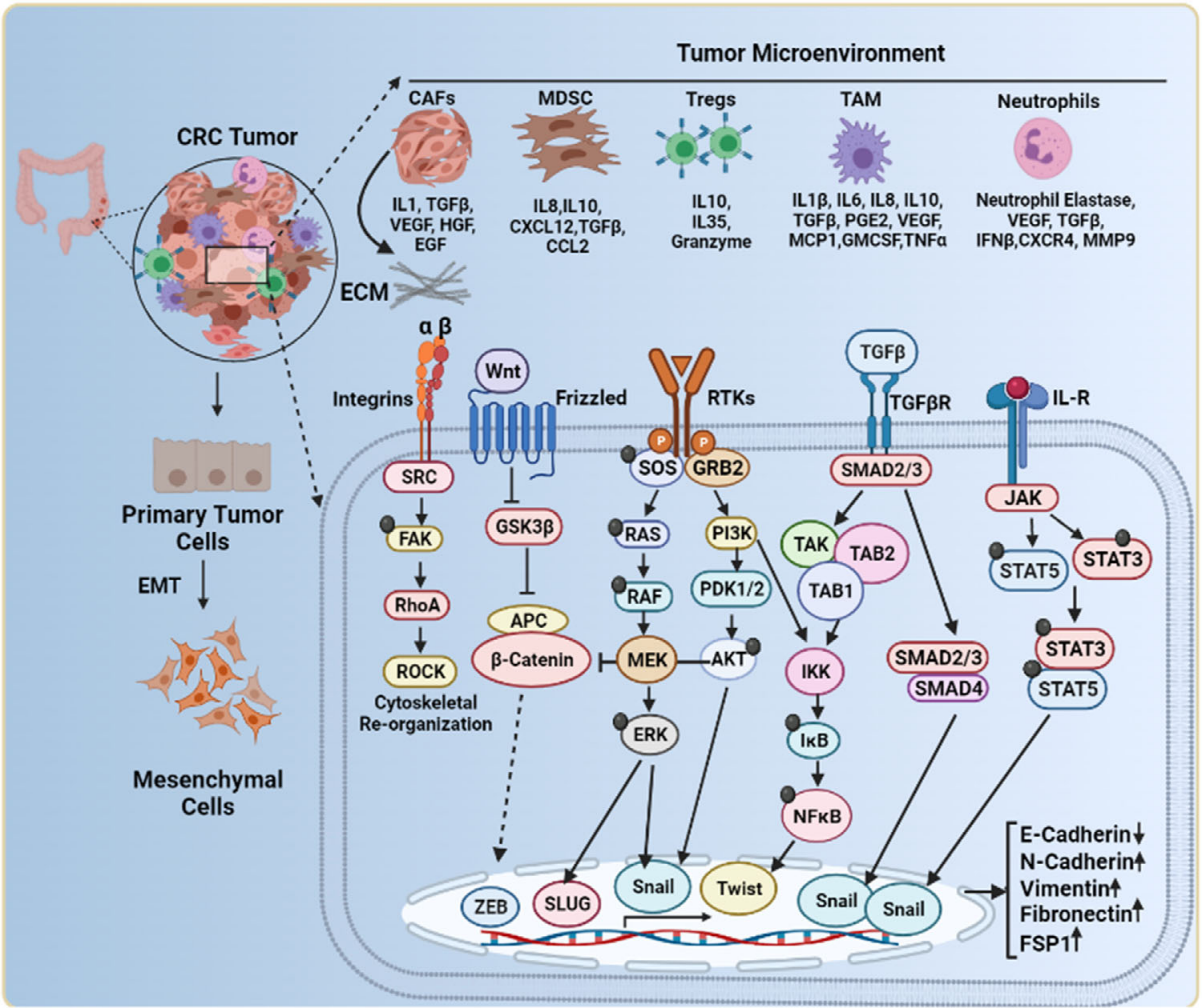
The complex network in the TME orchestrates EMT. CRC stromal cells such as Tregs, TAMs, MDSCs, CAFs, and neutrophils secrete various cytokines and chemokines into the surrounding environment. These soluble factors activate diverse signaling pathways, including Ras/Raf/ERK, PI3K/AKT, TGF/SMAD, JAK/STAT, Integrin/FAK, and Wnt/GSK/β‐catenin. Activation of these signaling pathways induces EMT in CRC cells by promoting a transcriptional program that downregulates E‐cadherin and upregulates N‐cadherin, vimentin, fibronectin, and FSP1. Integrin/FAK signaling pathway activation induces cytoskeleton reorganization, a prerequisite for invasion and metastasis. **Abbreviations**: Tregs, regulatory T cells; TAMs, tumor‐associated macrophages; MDSCs, myeloid‐derived suppressor cells; CAFs, cancer‐associated fibroblasts; EMT, epithelial to mesenchymal transition; CRC, colorectal cancer; FSP1, ferroptosis suppressor protein 1; Ras/Raf/ERK, rat sarcoma virus/ rapidly accelerated fibrosarcoma/ extracellular signal‐regulated kinases; PI3K/AKT, phosphoinositide 3‐kinases/ protein kinase B; TGF/SMAD, transforming growth factor/ mothers against decapentaplegic homolog 1; JAK/STAT, Janus kinase‐signal transducer and activator of transcription

Metastasis is a dynamic process through which cancer cells migrate from the primary tumor site to distant organs/tissues. CRC metastases primarily comprise peritoneal cavity metastases and liver metastases [154], which are the primary causes of cancer‐related morbidity and death in CRC [155]. The central phenomenon that enables immobile epithelial cells to gain mesenchymal properties as a prerequisite for migration and invasion is EMT (**Figure** 4). During EMT, cells lose cell‐cell adhesion and cell‐matrix adhesion, thereby gaining enhanced migration and invasion capabilities [156]. E‐cadherin is used as a common marker of EMT, and its downregulation is positively associated with lymph node metastasis, angiogenesis, and poor differentiation in CRC [157]. Expression of cadherin 1 (*CDH1*), the gene encoding E‐cadherin, in CRC is suppressed by various transcription factors: snail family transcriptional repressor 2 (SNAI2), zinc finger E‐box‐binding homeobox 1 (ZEB1), transcripton factor activating enhancer‐binding protein 4 (TFAP4), twist‐related protein 1 (TWIST1), transcription factor 4 (TCF4), SRY‐box transcription factor 2 (SOX2), octamer‐binding transcription factor 4 (OCT4), Nanog, high mobility group AT‐hook 1 (HMGA1), and fos‐related antigen 1 (FRA1) [157, 158, 159, 160, 161, 162, 163, 164, 165, 166]. *CDH1* transcriptional repressors, such as AP4, OCT4, and SOX2, are positively associated with liver metastasis in CRC [159, 161, 162].

*SNAI2* expression is markedly associated with vimentin (a mesenchymal marker), lymph node involvement, and poor prognoses in CRC [167]. *TWIST1* is also overexpressed in 85% of CRCs and is associated with lymph node positivity and poor survival [166, 167]. TNF‐α is associated with EMT by upregulating the activity of SNAI1 through the inactivation of the AKT‐glycogen synthase kinase 3 beta (GSK3β) axis [168]. Zinc finger protein SNAI1 (SNAIL) family members are activators of EMT and are highly expressed by CRC colonospheres containing colon cancer stem cells. Hwang *et al*. [169] showed that 227 SNAIL‐activated genes were upregulated in colonospheres with gene regulatory networks centered around IL‐8 and Jun in CRC. IL‐1β induces mesenchymal characteristics by positively regulating the canonical Wnt signaling pathway in CRC through GSK3β inhibition [170]. IL‐1β has been found to induce EMT in CRC cells through Zeb1 upregulation. The silencing of Zeb1 results in the reversal of IL‐1β‐induced EMT and stem cell characteristics [171]. IL‐6 triggers EMT via JAK2/STAT3 signaling in CRC cells [172]. The presence of IL‐6 in the conditioned media of human colon cancer‐derived mesenchymal stem cells enhances the migration and metastatic capability of CRC cells by upregulating Notch1 and CD44 levels [173]. IL‐11 is also the dominant STAT3‐activating cytokine and is involved in the progression of gastrointestinal tumors [174]. Furthermore, IL‐11R considerably decreases CRC tumorigenicity in dextran sodium sulfate/azoxymethane and *APC* ^min^ (heterozygous mutation in the adenomatous polyposis coli [*APC*] gene) mouse models. Moreover, IL‐6 inactivation only reduces the CRC burden in *APC* ^min^ mice [174]. IL‐11 secreted by CAFs, due to TGF‐β exposure, triggers glycoprotein 130 (GP130)/STAT3 signaling in tumor cells [175], thereby demonstrating the prometastatic role of IL‐11 in CRC. IL‐8 is one of the prognostic classifiers for predicting the OS of patients with metastatic CRC [176]. Furthermore, CAFs secreted IL‐6 was shown to induce IL‐6R and c‐MET expression on CRC cells, thereby promote STAT3 activation, upregulation of TWIST1, inducing EMT [177].

Among various chemokine signaling pathways, the CXCL12‐CXCR4 signaling axis plays a pivotal role in cancer metastasis [178]. Recently, D'Alterio *et al*. [179] showed that combination therapy with the CXCR4 antagonist peptide R and chemotherapeutic agents inhibits cell proliferation and reverses EMT in colon cancer cells, thereby highlighting the importance of CXCR4 inhibitors in the management of chemoresistant CRC tumors. In advanced‐stage CRC, Th17 inhibits migration of CTLs in tumor tissues by downregulating the expression of *CXCR3* via the IL‐17A/STAT3 axis.

Anti‐IL‐17A or stattic (a STAT3 inhibitor) administration rescues Th‐17‐induced inhibition of CTL recruitment in a CRC mouse model [180]. Recently, Liang *et al*. [181] demonstrated that tripartite motif containing 47 (TRIM47) promotes CRC proliferation and metastasis by directly binding to SMAD family member 4 (SMAD4) and promoting its degradation via ubiquitination. This loss of SMAD4 triggers CCL15 expression in CRC. Therefore, the TRIM47‐SMAD4‐CCL15 axis presents a potential target for metastatic CRC.

TGF‐β is a multifunctional cytokine that generally acts as a potential tumor suppressor in healthy colonic cells. However, the dysregulated TGF‐β pathway in cancer cells promotes the survival, invasion, and metastasis of CRC cells, thereby acting as an oncogene. The TGF‐β signaling pathway exhibits a paradoxical role in CRC [182, 183]. The TGF‐β signaling works primarily by activating SMAD (a canonical pathway) and non‐SMAD signaling pathways (a noncanonical pathway) to induce EMT in cancer cells [184, 185]. Moreover, many signaling cascades are involved in TGF‐β signaling in cancer for promoting tumor progression [186, 187, 188], including a novel signaling cascade of TGF‐β‐metastasis associated 1 (MTA1)‐SOX4‐enhancer of Zeste 2 polycomb repressive complex 2 subunit (EZH2) that drives EMT in CRC. Undoubtedly this signaling axis can be used as a novel potential therapeutic target for many cancers [186]. In addition, TGF‐β and TNF‐α signaling synergy in the presence of activated p38 and MAPK can promote a rapid morphologic alteration of the highly organized colonic epithelium to dispersed cells with a mesenchymal phenotype [187]. TGF‐β also stimulates the four and a half LIM Domains 2 (*FHL2*) gene, a potent inducer of EMT, by stimulating vimentin and matrix metalloproteinase‐9 (MMP‐9) expression and deregulation of E‐cadherin in a TGF‐β‐dependent and SMAD‐independent noncanonical signaling pathway [188].

An in vivo study reported a TGF‐β‐induced pro‐metastatic program to be associated with a high risk of CRC relapse [175]. Additionally, augmented TGF‐β signaling in adjacent stromal cells increases organ colonization efficiency by CRC cells [175]. Moreover, genomic studies identified an aggressive CRC subtype with a mesenchymal phenotype, with *TGF*‐*β1* as a central gene for this signature [189]. Consistent with these findings, inhibiting TGF‐β signaling impairs experimental CRC liver metastasis [190].

It was recently shown by Chiavarina *et al*. [191] that metastatic CRC cells that have the potential to metastasize lack mutations in TGF‐β receptor but possess frequent p53 mutations. The study found that the TGF‐β pro‐metastatic activity in CRC cells was mediated through TGF‐β‐induced protein ig‐h3 (TGFB1/beta ig‐h3). Increased levels of TGFB1 were found in CRC liver metastasis and in serum samples from untreated CRC patients compared to those receiving chemotherapy. Moreover, the expression of TGFB1 was found to be predominant in vimentin‐positive stromal cells [191]. TGFB1 was found to exert its pro‐metastatic function by affecting the crosstalk between tumor and endothelial cells, thereby promoting angiogenesis [191]. Thus, silencing of TGFB1 was found to significantly reduce liver metastasis formation in vivo and suppress angiogenesis in vitro [191]. Another study showed that the interaction of CAFs with conditioned media from epithelial colon cancer cells (HCT116) resulted in hyperactivated TGF‐β1 signaling leading to enhanced phosphorylation of SMAD2 and SMAD3 and increased expression of plasminogen activator inhibitor‐1 (PAI‐1) [192]. Moreover, the stimulation of CAFs with HCT116‐conditioned media resulted in two‐fold higher expression of MMP‐3 and MMP‐14 compared to the stimulation of CAFs by TGF‐β1 [192]. Thus, these studies suggest that oncogenic activation of TGF‐β provides a continuous growth stimulus to promote EMT in CRC.

## 5 CYTOKINE‐ AND CHEMOKINE‐MEDIATED IMMUNOSUPPRESSION PROMOTES CRC METASTASIS

**FIGURE 5:**
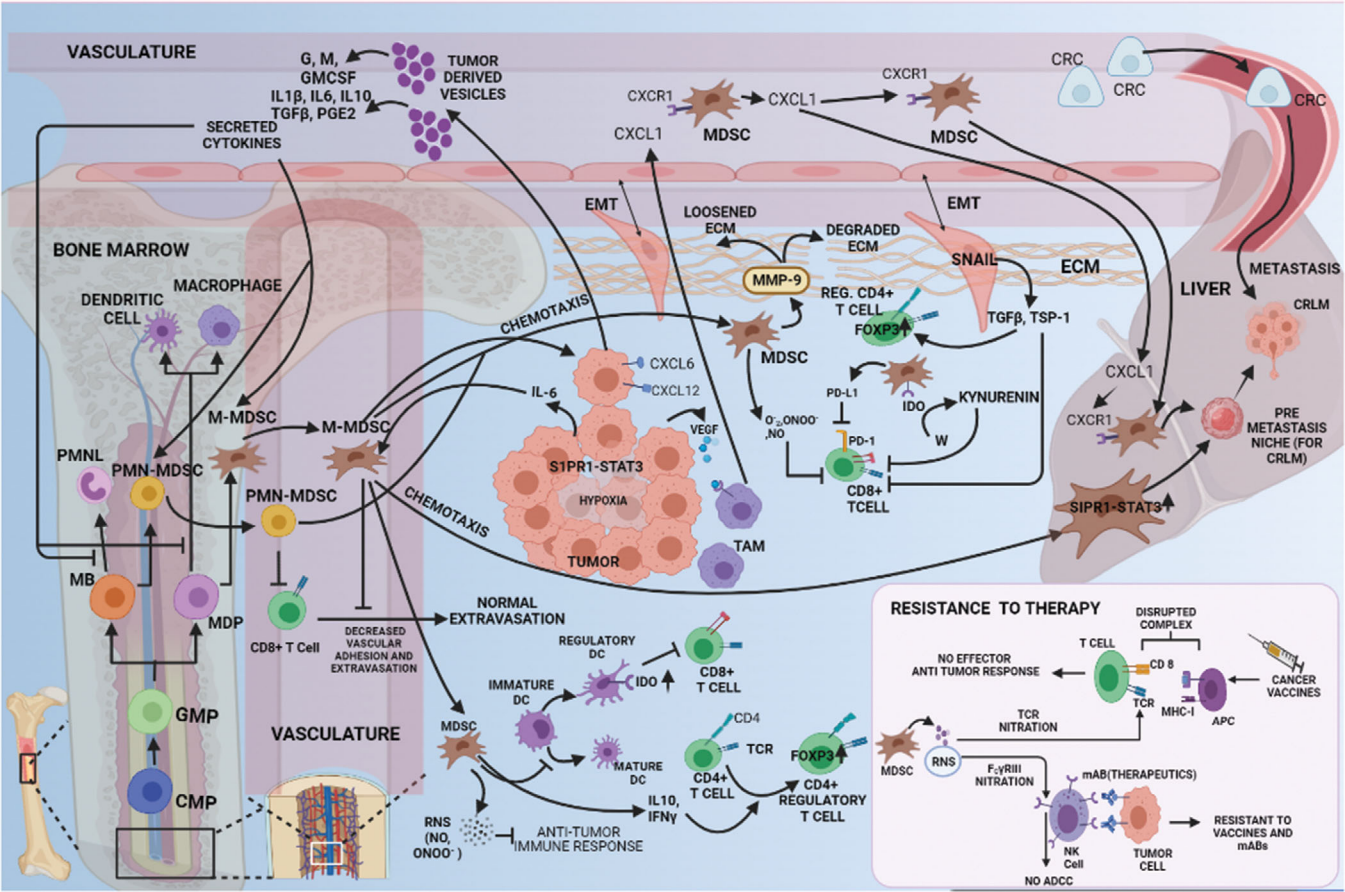
Cytokine‐ and chemokine‐mediated immunosuppression promotes CRC metastasis. Blunting antitumor immunity involves induction of regulatory DCs and CD4+ T cells and inhibition of circulating and tumor‐infiltrating CTLs through MDSCs (via RNS, IL‐10, IFN‐γ, PD‐L1, and IDO) and EMT‐exhibiting tumor cells (via TSP‐1). Tumor‐born cytokines trigger inflammation to interrupt myelopoiesis, yielding MDSCs that chemotactically reach the peritoneum or liver. RNS species also subvert immunotherapy by nitrating the TCR and CD16 to prohibit tumor‐directed cytotoxicity from NK/T cells. MDSCs mediate local invasion by MMP‐9 production. Tumor cells and TAMs direct circulating MDSCs to localize in the liver to forge the hepatic PMN needed for promoting LM. The most likely chemo‐attractants responsible for metastasis include IL‐6 from CRC cells exhibiting S1PR1‐STAT3 signaling and CXCL1 (in circulation or from premetastatic liver) from VEGFR‐engaged TAMs in the CRC TME. **Abbreviations**: CRC, colorectal cancer; DCs, dendritic cells; CTLs, cytotoxic T cells; MDSCs, myeloid‐derived suppressor cells; RNS, reactive nitrogen species; EMT, epithelial to mesenchymal transition; IL‐10, interleukin‐10; IFN‐γ, interferon gamma; PD‐L1, programmed death‐ligand 1; IDO, indoleamine‐pyrrole 2,3‐dioxygenase; TCR, T cell receptor; MMP9, matrix metalloproteinase 9; NK/T, natural killer/T; PMN, polymorphonuclear neutrophils; LM, liver metastasis; TAMs, tumor‐associated macrophages; IL‐6, interleukin‐6; S1PR1, sphingosine‐1‐phosphate receptor 1; STAT3, signal transducer and activator of transcription 3; CXCL1, C‐X‐C motif chemokine ligand 1; VEGF‐R, vascular endothelial growth factor‐receptor; TME, tumor microenvironment; CD16, cluster of differentiation 16

The importance of systemic inflammation in driving the prerequisites of cancer metastasis is well known. In addition to overcoming the physical obstruction of the tissue stroma by degradation of the ECM, systemic inflammation promotes immunosuppression, stem cell‐like properties, and organization of the PMN for successful organ‐specific homing of metastatic cells (**Figure** 5). The underlying mechanisms involve distinct cellular and molecular mediators from within the tumor and outside. In support of the tumor‐specific effects, Kudo‐Saito *et al*. [193] showed that *SNAI1*‐expressing tumor cells induce local immunosuppression by the production of TGF‐β and thrombospondin‐1 (TSP‐1). These molecules hamper the differentiation of immature DCs and CD4**^+^** T cells, with concomitant upregulation of master transcription regulator FOXP3 and immune checkpoint inhibitors, such as and programmed cell death protein 1 (PD‐1) and cytotoxic T‐lymphocyte‐associated protein 4 (CTLA‐4). This, in turn, induces Tregs and regulatory DCs and decreases the proliferation of local CTLs [193]. Furthermore, cytokines such as GM‐CSF, macrophage colony‐stimulating factor (M‐CSF), IL‐6, IL‐10, IL‐1β, PGE2, and TGF‐β, whether secreted or produced by tumor‐derived exosomal vesicles, enter the bone marrow or circulation, and generate MDSCs [194]. Chronic inflammation also compromises myelopoiesis by impairing myeloid progenitor differentiation to polymorphonuclear leukocytes and monocytes and generating protumor immature myeloid cells [194].

CRC MDSCs comprise two distinct populations: lineage negative‐human leukocyte antigen‐DR isotype (Lin**^−^** HLA‐DR**^−^**)/low CD11b**^+^** CD14**^−^** CD15**^+^** CD33**^+^** for PMN‐MDSCs and Lin**^−^** HLA‐DR**^−^**/low CD11b**^+^** CD14**^+^** CD15**^−^** CD33**^+^** for monocytes [195]. Both immature myeloid cells and MDSCs exacerbate metastasis via immunosuppression, PMN formation, stem cell‐like properties, and vascular destabilization [196]. In addition to producing MMP‐9 to degrade ECM components and facilitate stromal invasion [195, 196], MDSCs also produce cytoinhibitive and cytolytic reactive oxygen species (ROS) and reactive nitrogen species (RNS), including superoxide, peroxynitrite, nitric oxide, hydroxyl radicals, and peroxides [194]. These reactive species facilitate MDSC accumulation in the TME and deplete arginine, induce indoleamine 2,3‐dioxygenase (IDO)‐mediated tryptophan degradation, and sequester cysteine. This then induces CTL anergy/apoptosis [194], elevates expression of CTLA‐4 and PD‐1, and causes resistance to immunotherapy [194]. The underlying mechanisms of CTL anergy/apoptosis include T‐cell receptor (TCR) nitration [194] that alters conformation, disruption of TCR‐major histocompatibility complex class 1 (MHC1) complex formation, and antigen presentation [197]. In addition, nitration of NK cell receptor immunoglobulin G Fc Receptor 3 (FCγR3) suppresses the opsonization of antigen‐bearing cells to elicit antibody‐dependent cytotoxicity [198]. Although this phenomenon confers resistance to cancer vaccines [199], RNS/ROS inhibitors combined with immunotherapeutic agents reverse this tolerance [197]. In addition, Treg and TAM induction or prevention of T‐cell infiltration via decreased vascular adhesion supports an immunosuppressive TME, rendering the tumor cells more resistant [194].

CRC‐related systemic inflammation creates an immunosuppressive TME and forms the PMN in distant organs [196]. IL‐6‐mediated MDSC induction and recruitment induces the PMN in the liver via the sphingosine‐1‐phosphate receptor 1 (S1PR1)‐STAT3 signaling network [200]. Although this suggests that the IL‐6‐S1PR1‐STAT3 signaling is a promising therapeutic target [200], how IL‐6 recruits MDSC specifically to the liver remains elusive. VEGFA secreted by CRC cells also creates a PMN in the liver by stimulating TAMs and producing CXCL1 [201]. Liver CXCL1 chemotactically recruits CXCR2**^+^** MDSCs to configure the tissue architecture that ultimately promotes liver metastasis [201]. Increased CXCL1 levels also recruit neutrophils to the liver, which establishes the PMN [202].

Interestingly, inhibiting the CXCL1 receptor CXCR2 with a function‐blocking antibody inhibits liver metastasis in an orthotopic mouse model of CRC [202]. In addition, the anti‐angiogenic receptor tyrosine kinase inhibitor TSU68 suppresses *CXCL1* expression in the liver PMN, which is associated with decreased CXCR2^+^ neutrophil infiltration and subsequent liver metastasis, in a mouse model of CRC [202]. Ablation of the CXCL8‐CXCR2 axis in *CXCR2*‐knockout mice impairs CRC growth, migration, and metastasis [103]. Furthermore, CXCR1 and CXCR2 antagonists inhibit CRC liver metastasis by decreasing angiogenesis and inducing tumor cell apoptosis in vivo [105]. Mouse‐derived CRC cells expressing *CCL9* recruit CCR1**^+^** myeloid cells (neutrophils, eosinophils, monocytes, and fibrocytes) to promote liver metastasis [203]. Loss of *SMAD4* induces *CCL15* (i.e., human ortholog of mouse CCL9) expression [181] and promotes CCR1**^+^** MDSC recruitment and development of CRC liver metastasis [204]. Importantly, patients with CRC and CCL15 protein expression in liver metastasis experience reduced relapse‐free survival than those with CCL15^–^ CRC [204]. Similarly, CX3CL1‐CX3CR1 recruits TAMs to the CRC TME and promotes liver metastasis [205]. Intrasplenic injection of SL4 cells in *CX3CR1*‐knockout mice decreases liver metastases and is associated with reduced VEGF, TGF‐β, CCR2, CXCR4, and VEGFR2 levels [205].

CCL2 (MCP‐1) protein levels are elevated in CRC and are an accurate predictor of liver metastasis [206]. CCL2‐CCR2 is a potent recruiter of TAMs that promotes tumor growth, progression, and metastases. In an allograft mouse model, *CCL2*‐expressing CRC cells recruit CCR2**^+^** CD11b^+^ Gr1^mid^ myeloid cells and promote liver metastasis [207]. CAF has also been shown to promote immunosuppressive CRC TME by upregulating the secretion of CCL2, recruiting myeloid cells, decreasing T‐cell activity [208], and increasing PD‐L1 expression [209]. More recently, melanoma cell adhesion molecule (MCAM)‐positive CAFs have also been shown to promote immune suppressive CRC TME by increasing NF‐κB‐IL34/CCL8 signaling via interleukin‐1 receptor 1 interaction and promoting macrophage infiltration [210]. Similar CD11b**^+^** CCR2**^+^** cells are present in CRC liver metastasis foci. Increased *CXCR4* expression in the hypoxic conditions of CRC [211] is associated with tumor relapse, liver metastasis, and poor prognosis [212, 213]. Although CXCL12, a ligand for CXCR4, acts as a tumor promoter [87] or tumor suppressor [86], impairment of the CXCL12‐CXCR4 signaling axis suppresses CRC liver metastasis [214]. In support of this, Kaplan *et al*. [215] also demonstrated that CXCL12 produced by bone marrow‐derived cells attracts CXCR4**^+^** cancer cells to the PMN. CCL20 [100] and CCR6 levels [216] are upregulated in primary CRC tumors and serum samples, respectively, and are associated with liver metastasis [217]. Although the underlying mechanism is completely unknown, recruitment of CCR6**^+^** Tregs by TAMs releasing CCL20 in the liver is considered the most likely mechanism [218]. Like CXCL12, CXCL9/10 recruits CXCR3**^+^** CRC cells to the lymph nodes [219], and patients with active CXCL10‐CXCR3 signaling axis function have increased metastases [220]. Importantly, patients with CXCR3**^+^** tumors have considerably lower survival rates than patients with CXCR3**^–^** CRC [219]. Similarly, elevated CCL21‐CCR7 signaling axis function upregulates MMP‐9 protein expression, increases lymph node metastases [221], and is associated with poor OS in patients with CRC [222]. In contrast, increased CCR7 levels in CRC cells improve the 3‐year survival rate [223].

Peritoneal cavity metastases develop in most patients with CRC, which is a substantial cause of death. Although CRC peritoneal cavity metastases is considered the terminal stage of locally advanced disease and CRC liver metastasis is regarded as a hematogenous disseminating disease, the mechanisms of organotropism and dissemination routes are only now becoming elucidated. Peritoneal macrophages, mesothelial cells (PMCs), and fibroblasts play an essential role in the development of peritoneal cavity metastases [224]. Peritoneal macrophages highly express CXCR4, VEGF, TGF‐β, and ICAM‐1 proteins, and increased levels of CXCR4, VEGF, and ICAM‐1 are associated with CRC peritoneal cavity metastases [225]. PMCs prevent initial tumor cell invasion by secreting TNFα, CRC cells secrete IL‐6, and IL‐10 converts these anti‐tumorigenic PMCs to a pro‐tumorigenic M2 phenotype favoring tumor progression [226]. Like CAFs, M2 PMCs secrete cytokines, chemokines, growth factors, ECM components, and adhesion molecules into the TME, facilitating peritoneal cavity metastases development [226]. The peritoneal cavity metastases TME increases the numbers of IFN‐γ cells, CXCR5**^+^** follicular helper T cells, CD20**^+^** B cells, and CD79a**^+^** and CD138**^+^** plasma cells [227]. Furthermore, increased levels of IL‐15, IL‐17, VEGFA, CD34, runt‐related transcription factor 1 (RUNX1), TGF‐β1, and TGF‐β3 also occur in the peritoneal cavity metastases TME [227]. In addition to PMCs, a small subpopulation of CD90**^+^** CD45^–^ mesothelial‐like cells express collagen I, alpha‐smooth muscle actin (α‐SMA), and vimentin, promote PMN, and support peritoneal cavity metastases development [228]. In support, intraperitoneal injection of mesothelial‐like and CRC cells promotes tumor growth and peritoneal cavity metastases in vivo [228]. Overall, these discoveries illustrate the importance of inflammation‐mediated organ‐specific metastasis, immunotherapy resistance, and tumor aggressiveness, and the development of novel therapeutics against inflammation in CRC.

## 6 CYTOKINE SIGNALING AS A THERAPEUTIC INTERVENTION IN CRC

Identifying a promising therapeutic target in CRC is challenging because of the complex interactions between cytokines in different networks. However, recent studies have indicated that targeting cytokine signaling may improve chemo‐immunotherapeutic responses in CRC. For example, IL‐17A exhibits a dual effect in *IL‐17A*‐deficient mouse models of hereditary polyposis. It reduces the number of polyps and promotes the expansion of RORγt Tregs and tumor invasion [63]. Although inhibiting IL‐17A reduces intestinal inflammation to some extent, inhibiting IL‐22 markedly reduces dysplasia and tumor burden in a mouse model of CRC [229]. Indeed, early epithelial barrier loss due to activation of β‐catenin or loss of *APC* and activation of IL‐17/IL‐23‐driven inflammation elicited by tumors governs CRC development and progression. Therefore, IL‐17/IL‐23 expression may be used as a potential therapeutic target for the treatment of CRC [24]. Likewise, the expression of IL‐6 and IL‐6R plays an essential role in CRC pathogenesis [230]. IL‐6 is involved in recruiting immune cells, producing proinflammatory cytokines, and modulating Th17 and Treg cells in CRC [44].

Because cancer cells depend on aerobic glycolysis for increased growth and proliferation, treatment with human recombinant IL‐6 stimulates aerobic glycolysis and promotes the proliferation of SW480 and SW1116 colon cancer cells [231]. Interestingly, the tumor‐promoting effect of IL‐6 is inhibited by the knockdown of the gene encoding 6‐phoshofructo‐2‐kinase/fructose‐2,6‐bisphosphatase‐3 [231]. Moreover, CRC tumor‐derived IL‐6 increases the phagocytic potential and migration of THP‐1 cells and human monocytes through STAT3 phosphorylation, thus suggesting that IL‐6 secretion by CRC cells can increase the phagocytic and migratory potential of macrophages in the TME and serve as a therapeutic option for CRC treatment [232]. Interestingly, simultaneous neutralization of IL‐6 and IL‐22 (i.e., STAT3 signaling drivers) and both TNF‐α and IL‐17A (i.e., NF‐κB signaling drivers) reduces the proliferation of human CRC cell lines treated with culture supernatants of isolated TILs [35]. Indeed, intravenous injection of the STAT3 inhibitor BP‐1‐102 into a mouse model of sporadic CRC decreases TIL‐induced activation of NF‐κB and STAT3 and thus CRC cell proliferation [35]. The central regulatory pathways downstream of IL‐6 may also be targeted in combination therapies for CRC. IL‐6 secretion is accompanied by increased Fos‐related antigen 1 deacetylation transcriptional activity, leading to cancer stem cell‐like properties and tumor progression associated with poor prognosis. Interestingly, 5‐fluorouracil and tubastatin A, a selective histone deacetylase 6 inhibitor, synergistically prevent tumor growth by blocking CRC stem cell‐like properties [233].

Emerging studies indicate that the IL‐33/ST2 axis may represent a new therapeutic target in CRC. Indeed, increased IL‐33/ST2 levels and increased IL‐33**^+^** and ST2**^+^** microvessel densities occur in the stroma of adenomas and CRCs, which suggests a contributing role of the IL‐33/ST2 pathway to CRC pathogenesis [146]. Similarly, IL‐33 contributes to the formation of a protumorigenic environment by promoting intestinal polyposis through the activation of stromal cells (i.e., subepithelial myofibroblasts and mast cells) in an *Apc^Min/+^* mouse model [234]. Blocking IL‐33 signaling reduces polyp growth, increases apoptosis, and inhibits angiogenesis in *Apc^Min/+^* mice, thereby showing the potential of IL‐33 as a therapeutic target in CRC [234]. Interestingly, neutralizing the IL‐33/ST2 pathway improves colitis and enhances mucosal healing in IBD mouse models [235]. Furthermore, IL‐33 is a reported influential proangiogenic factor in CRC [236, 237] and promotes angiogenesis and vascular leakage by stimulating endothelial nitric oxide triggered by the ST2/TNF receptor associated factor 6 (TRAF6)‐Akt‐endothelial nitric oxide synthase (eNOS) signaling pathway [236]. In support of this, the soluble form of the IL‐33 receptor inhibits angiogenesis induced by IL‐33 and thus malignant growth in mouse and human CRC cells [237]. Although most studies have reported IL‐33 as a tumor promoter, conflicting studies have described the antitumor effects of IL‐33 in colon cancer [148], melanoma [238], and hepatocellular carcinoma [239]. IL‐33, therefore, plays a complex role in CRC development. More exploratory studies are needed to elucidate the exact role IL‐33 plays in CRC and subsequently develop therapeutic interventions that target IL‐33/ST2 signaling [147]. The critical involvement of MDSCs in CRC aggressiveness has also revealed MDSCs as important therapeutic targets in CRC [194]. IL‐6‐S1PR1‐STAT3 signaling pathway inhibitors can prevent liver metastasis development and improve patient prognosis [194]. In addition, administration of ROS/RNS inhibitors, along with chemo‐immunotherapeutics, improves responses in CRC [197, 198].

*KRAS*, one of the most critical oncogenes in colon cancer, is also emerging as a potential therapeutic target for CRC treatment. Many studies have reported an essential role of KRAS signaling in tumorigenesis and inflammation [240, 241, 242]. *KRAS*‐mutant cancer cells modulate the properties of CAFs, endothelial cells, and ECM composition [240]. In CRC development, the differential expression of IL‐17, IL‐22, and IL‐23 is associated with CRC progression in patients with *KRAS* mutations [243]. IL‐17, IL‐23, and GM‐CSF mRNA and protein levels are higher in KRAS^+^ CRC tissues. In contrast, IL‐22 levels are higher in KRAS^–^ CRC tissues [243].

In comparison, IFN‐γ levels are lower in CRC tissues (KRAS^+^ or KRAS^–^) than in healthy tissues. Furthermore, treatment with the KRAS inhibitor manumycin A inhibits cell viability and increases apoptosis in a dose‐dependent manner in Caco2 cells [243]. The findings that cytokine expression is influenced by KRAS signaling and mutations in *KRAS* are tightly linked to tumor‐promoting inflammation suggest a molecular link between inflammation and tumorigenesis and a viable therapeutic option for KRAS targeting.

An essential modulator of the inflammatory processes in IBD is the transcription factor NF‐κB [244]. NF‐κB is essential in maintaining intestinal homeostasis as it functions in controlling epithelial integrity and interaction between the mucosal system and gut microflora [245]. NF‐κB has been reported to be activated in the macrophages and epithelial cells of inflamed mucosa from patients with ulcerative colitis, Crohn's disease, and non‐specific colitis [246]. Canonical activation of the NF‐κB pathway is triggered by fungal, microbial, and viral byproducts and a variety of proinflammatory cytokines that activate the IκB kinase (IKK) complex [247]. It has been reported that the deletion of IKKβ in the intestinal cells failed to reduce inflammation but lead to a significant decrease in tumor incidence without affecting the tumor size in colitis‐associated cancer (CAC) mouse model [248]. However, the deletion of IKKβ in myeloid cells significantly decreased the tumor size, thus demonstrating that the specific inactivation or pharmacological inhibition of IKKβ in different cell types can effectively treat CAC [248]. Another mouse model study showed that the specific inhibition of NF‐κB through the conditional ablation of NF‐κB essential modulator (NEMO) in the intestinal epithelial cells led to severe colitis and compromised epithelial integrity and bacterial translocation [245]. Moreover, the study showed that NEMO deficiency sensitized the intestinal epithelial cells to TNF‐induced apoptosis, suggesting that active TNF signaling causes colonic inflammation and disrupts the epithelial barrier integrity by killing the NF‐κB‐deficient intestinal epithelial cells [245]. TNF‐α is another potent cytokine that is implicated in various chronic inflammatory diseases, including IBD, due to its central role as a key regulator of inflammatory responses [249]. Preclinical studies have shown the involvement of TNF‐receptor 2 (TNFR2) signaling in the development of CAC, suggesting that TNFR2 activation is an important mechanism for epithelial barrier loss, NF‐κB‐dependent tumor cell survival, and proinflammatory cytokine release [250, 251, 252]. Another preclinical study showed that treatment of wild‐type mice with azoxymethane and dextran sulfate sodium increased the expression of TNF‐α and infiltrating leukocytes in the lamina propria and submucosal colonic regions leading to the formation of multiple colonic tumors [253]. Moreover, the study showed that transplanting the wild‐type mice with TNF‐receptor p55 (TNF‐Rp55)‐deficient bone marrow and treatment with TNF‐α antagonist reduced mucosal damage and colonic infiltration by macrophages and neutrophils [253].

Thus, TNF‐NF‐κB signaling has been identified as an essential regulatory signaling pathway that has a substantial role not only in tumor initiation and promotion but also as an important modulator of intestinal homeostasis. Many studies have shown the involvement of these proinflammatory cytokines in the pathogenesis of IBD, but their role in CAC is limited to preclinical studies. Given the potent role of these proinflammatory cytokines in inflammation and tumorigenesis, they can serve as promising therapeutic targets and can provide a mechanistic insight that elucidates their exact roles in the pathogenesis of CAC.

The requirement of impaired immune cell differentiation for successful metastasis and tumor aggressiveness suggests that the design of therapeutic agents should not only recruit immune cells to the TME but also ensure their lasting functional activation. Further clinical studies are needed to explore the clinical efficacy of targeting cytokines individually or multiple cytokines signaling pathways as a combinatorial therapeutic approach for CRC. Specifically, using JAK inhibitors, monospecific agents, and bispecific antibodies to attack and neutralize signaling from several cytokine pathways represents therapeutic strategies that may be useful for treating CRC. Comparative studies of transformed versus non‐transformed cells in vitro and in vivo are needed to assess the safety of targeting different cytokine/chemokine networks in CRC.

## 7 THE PROMISE OF IMMUNOTHERAPY IN CRC

Classical CRC treatment regime includes chemotherapy, irradiation, and surgery. However, immunotherapy has evolved as a breakthrough for CRC treatment. Immunotherapy has become a mainstay for the treatment of several cancers, and there are several US Food and Drug Admiration (FDA)‐approved CRC immunotherapy options available for tumors with high microsatellite instability (MSI‐H) or DNA mismatch repair deficiency (dMMR) [254]. Targeted monoclonal antibodies like Bevacizumab (Avastin®), which targets the angiogenic VEGF/VEGFR pathway, have been approved as first‐line therapy alone as well as in combination with other chemotherapeutic drugs for patients with advanced CRC [255]. Ramucirumab (Cyramza®) is another monoclonal antibody targeting the VEGF/VEGFR2 pathway that has been approved for patients who had failed the first‐line therapy following a phase III RAISE trial [256].

The epidermal growth factor receptor (EGFR) signaling pathway is an essential pathway that is involved in the proliferation of cancer cells, and monoclonal antibodies against EGFR like cetuximab (Erbitux®) and panitumumab (Vectibix®) have been used as a monotherapy in patients with wild‐type KRAS and NRAS tumors [257]. In a phase III clinical trial for CRC, it was observed that patients treated with cetuximab alone had a response rate of about 11%, but treatment with cetuximab in combination with irinotecan increased the patient response rate to around 17.5‐29.1% [258]. Similar results were reported for panitumumab as well [259]. The location of the tumor and its corresponding TME in CRC has a particular impact on the type of immunotherapy being administered. It was reported that bevacizumab treatment in patients with right‐sided tumors resulted in a prolonged survival (24.2 months) than those treated with cetuximab (16.7 months). The inverse was seen with left‐sided tumors, where cetuximab was associated with increased OS (36 months) compared with bevacizumab (31.4 months) [260]. This study provided solid evidence for the relation of TME with immunotherapy.

Immune suppression mechanisms mediated by T cells is one of the well‐studied mechanisms used by cancer cells to escape immune surveillance by rewiring cytokine networks that promote Tregs and MDSCs to inhibit cytotoxic T cell function [126]. CRC tumors also upregulate immune checkpoint molecules like programmed death‐ligand 1 (PD‐L1), which results in peripheral T cell exhaustion leading to reduced apoptosis in tumor cells [261]. An upregulation of immune checkpoint inhibitors such as PD‐1 and PD‐L1 has been reported in microsatellite instability high‐deficient mismatch repair (MSI‐HdMMR) CRC tumors seen in early‐stage cancers which allow immune evasion by TILs [262, 263]. As of today, there are two US FDA‐approved immune checkpoint inhibitors targeting PD‐1 for use in dMMR and MSI‐H advanced CRC patients who have failed the first‐line therapy [264]. Another checkpoint inhibitor, pembrolizumab (Keytruda®) that targets the PD‐1/PD‐L1 pathway, was approved for a subset of patients with advanced CRC having MSI‐H, dMMR, or high tumor mutational burden [265]. In another trial, patients were administered nivolumab (Opdivo®), a checkpoint inhibitor that targets the PD‐1/PD‐L1 pathway, and it was approved to be used alone or in combination with ipilimumab for a subset of patients with advanced CRC that has MSI‐H [266, 267]. Collectively, the data from all these trials highlight how detailed understanding of the TME is essential for the development of immunotherapy, which has tremendously improved objective outcomes in CRC patients who have deficient MMR.

## 8 TUMOR HETEROGENEITY IN CRC AND ITS IMPACT ON CANCER THERAPEUTICS

As reported by several studies, the pathological landscape of CRC is found to be very complex and molecularly heterogenous. Modern high‐throughput sequencing of CRC genome has revealed that nearly 16% of CRCs have high tumor mutational burden caused by defective DNA MMR that leads to the accumulation of mutations in microsatellites leading to the MSI phenotype [268, 269, 270]. The remarkable genomic and epigenomic instability drives different oncogenic molecular pathways and contributes to the tumor heterogeneity in CRC [271]. Tumor heterogeneity can be further classified to inter‐tumor (tumor by tumor) heterogeneity, which refers to the presence of genetic alterations in different tumors from a single patient, and intra‐ tumor (within a tumor) heterogeneity, in which different clones of cancer cells can be found within the same tumor from a single patient, thus posing a major challenge for CRC treatment. It is now well established that even tumors at different locations exhibit different genetic signatures with right‐ and left‐sided tumors having distinct genetic profiles [272]. Right‐sided tumors generally develop in patients with genetic predisposition to CRC and display a high frequency of *BRAF* mutation as well as MSI. While left‐sided tumors exhibit extensive chromosomal instability (CIN) and a gene expression profile that involves EGFR pathway activation [85, 273]. In relation to the TME, increased expression of endothelial nitric oxide synthase (eNOS), COX2, and ephrin type‐B receptor 4 (EPHB4) was observed in right‐sided tumors. While the systemic immune‐inflammation index, neutrophil‐to‐lymphocyte ratio, and the platelet‐lymphocyte rate were found to be higher in left‐sided tumors [274]. In addition to inter‐tumor and intra‐tumor heterogeneity, CRC is also characterized by spatial heterogeneity which refers to the co‐existence of different cancer cell types (differentiated and undifferentiated) within the same tumor and also the co‐existence of genetically distinct clones in a single metastatic lesion [275]. In addition, metastatic lesions are often associated with drug refractory clones [276]. The origin of different types of clones has been attributed to immune cell heterogeneity which is influenced by localized tumor cells, stroma, microenvironmental factors and composition of the gut microbiota [277]. A study by Kreso *et al*. [278] showed the functional heterogeneity of individual tumor cells within a uniform genetic lineage in CRC. The study performed copy number alteration profiling and mutational analysis to distinguish individual clones and found the clones to be stable after serial transplantation. Despite the stability, the lentiviral clones showed intra‐clonal heterogeneity that affected response to chemotherapy and contributed to tumor growth.

In another study, authors looked upon the genetic heterogeneity among CRC patients with liver metastases and its relationship with chemotherapy exposure and patient outcome [279]. While they observed substantial variation in the level of intra‐patient and intra‐tumor heterogeneity, heterogeneity was higher in patients that were previously exposed to chemotherapy. Patients with a low level of heterogeneity had an higher OS rate compared to patients with a high level of heterogeneity [279]. Thus, these studies show that spatial heterogeneity provides substrates for the emergence and evolution of drug resistance and poses a challenge for the successful treatment of CRC. Detailed discussion of CRC tumor heterogeneity is beyond the scope of current review and has been covered in many excellent reviews to which readers can refer [275, 280].

## 9 CONCLUSIONS AND FUTURE PERSPECTIVES

Cytokine networks contribute to tissue homeostasis in the gut and are critical mediators of inflammation and oncogenesis. Cytokine networks function as a double‐edged sword that positively influences antitumor immunity and tumorigenic inflammation. The protumorigenic or antitumorigenic functions of each cytokine are influenced by crosstalk in the complex cytokine environment. The balance between these two opposite inflammatory networks in the tumor stroma and vasculature may decide the course of CRC development. The inflammatory cytokines generated under nonspecific conditions by several different cell types such as CD4**^+^** T cells, innate lymphoid cells, and TAMs may further cause CAFs to generate cytokines and growth factors that promote apoptosis resistance, aberrant growth, proliferation, angiogenesis, invasiveness, and metastasis, which are all critical hallmarks of cancer. Despite the significant evidence of tumor‐promoting roles of CAFs in CRC, recent studies have shown tumor suppressive roles in many cancers. In CRC, although tumor‐promoting roles of CAFs have made them attractive therapeutic targets, surprisingly, complete depletion of CAFs have developed more aggressive tumors, suggesting their subtype‐based heterogeneous roles. While heterogeneity in CAF population can be the underlying reasons for mixed outcomes of CAF‐based therapeutics for CRC patients, future studies should be focused on better understanding of CAF pathobiology for improved efficacy and patient prognosis.

The studies cited in this review demonstrate that deregulated expression of specific cytokines and their receptors are related to various aspects of metastatic progression and aggressiveness of CRC and provide an overview of the underlying mechanisms. For example, TNF‐α induces EMT in CRC, thereby supporting metastasis via increased SNAI1 transcription factor activity. TNF‐α is also involved in AKT activation and repression of GSK‐3β, resulting in the stabilization of SNAI1. Two critical intracellular signaling pathways, STAT3 and NF‐κB, which are regulated by major cytokines and intestinal epithelial cell expression of their cognate receptors, contribute to multiple oncogenic functions essential to the EMT phenomenon. In the STAT3 signaling pathway, cytokine receptor interaction activates Janus kinases and tyrosine kinase 2. In the NF‐κB pathway, they recruit downstream adaptor molecules. Therefore, understanding the molecular networks that underlie inflammation‐mediated immunosuppression, EMT, their functional relation to malignant tumor development, and metastasis is important. Thus, cytokine‐ and chemokine‐mediated immunosuppression and the EMT mechanism appear to be an integral component of CRC metastasis. Detailed analysis of these components will improve prognosis and lead to novel treatment strategies. In addition to targeting intracellular signaling pathways, cytokines or their receptors serve as potential targets for many monoclonal antibodies, some of which are under investigation in clinical trials. To improve the efficacy of cytokine therapy, targeting multiple and distinct cytokine signal pathways or shared receptors by several monospecific or bispecific antibodies will be beneficial. This is currently an underexplored opportunity for CRC treatment research but may become more prevalent in the future.

In conclusion, complex cytokine networks regulate metastasis in PMNs and cooperate in the inflammatory TME to exert their tumorigenic effects. Although cytokine signaling is essential in primary tumors, targeting cytokine networks that cause relapse will be vital for acting directly within the PMN to prevent tumor progression in patients with CRC.

## DECLARATIONS

  

## COMPETING INTERESTS

The authors declare that they have no competing interests.

## AUTHORS' CONTRIBUTIONS

AAB, MS, BA, SN, TM, CPP, AS, SM, and TK wrote the manuscript and generated figures. MAM, AAB, and MH contributed to the concept and design and critically edited the manuscript. SH, SBY, PB, RR, MPF, SU, and PD performed critical revision and editing of the scientific content. All authors read and approved the final manuscript.

## ETHICAL APPROVAL AND CONSENT TO PARTICIPATE

Not Applicable.

## CONSENT FOR PUBLICATION

Not Applicable.

## AVAILABILITY OF SUPPORTING DATA

Not Applicable.
